# “Nothing’s actually happened to *me*.”: the experiences of fathers who found childbirth traumatic

**DOI:** 10.1186/s12884-017-1259-y

**Published:** 2017-03-07

**Authors:** Jody Etheridge, Pauline Slade

**Affiliations:** 1 Alder Hey Children’s NHS Foundation Trust, Eaton Road, West Derby, Liverpool, L12 2AP UK; 2 Institute of Psychology Health and Society, Whelan Building, The Quadrangle, Brownlow Hill, Liverpool, L69 3GB UK

## Abstract

**Background:**

Given the limited research into men’s experiences of being present at childbirth this study explored the experiences of fathers who found childbirth traumatic. The aim of the research was to investigate how men coped with these experiences; the impact on their lives; and their views on what may have helped to reduce distress.

**Methods:**

Participants were recruited via websites relating to birth trauma and parenthood. A consent and screening questionnaire was used to ensure that participants met the inclusion criteria of: being resident in the UK; being 16 years or older; having been present at the birth and answering yes to the question “At some point during the childbirth I experienced feelings of intense fear, helplessness or horror”. Semi-structured telephone interviews were completed with 11 fathers who reported finding childbirth traumatic. Participants also completed the Impact of Event Scale as a measure of trauma symptoms. Template Analysis was used to analyse the interview data.

**Results:**

Childbirth was experienced as “a rollercoaster of emotion” because of the speed and unexpectedness of events. Men described fears of death, mirroring their partner’s distress; trying ‘to keep it together’ and helplessly watching a catastrophe unfold. Fathers felt themselves abandoned by staff with a lack of information. Men were subsequently distressed and preoccupied with the birth events but tended to feel that their responses were unjustified and tried to cope through avoidance. Men described the need for support but reluctance to receive it.

**Conclusions:**

Fathers may experience extreme distress as a result of childbirth which is exacerbated by aspects of current maternity care. Maternity services need to be aware of the potential impacts of fathers’ attendance at childbirth and attend to fathers’, as well as mothers’, emotional responses.

## Background

Men are susceptible to a range of mental health problems following the birth of a child [1]. Paternal mental health difficulties have been shown to increase the risk of emotional and behavioural problems in children [2] and affect the relationship with the partner [3]. Research into men’s mental health following childbirth has focused primarily on post-natal depression [1]; however, anxiety may be the most common post-natal mental health problem experienced by new parents [4].

Up to seven percent of women may experience symptoms of post-traumatic stress after childbirth, with prevalence of full Post-Traumatic Stress Disorder (PTSD) estimated at between 3 and 6% [5]; subjective distress during labour and obstetric emergencies being the most significant risk factors [6]. Post-traumatic stress symptoms in men following childbirth is a less explored area of research. Consequently the prevalence of PTSD after childbirth in men is difficult to quantify; with studies finding symptoms in between zero and five percent of the populations sampled between 6 and 9 weeks post-partum [7, 8]. However, studies of fathers have relied on questionnaire measures and have failed to assess the full diagnostic criteria for PTSD [9].

Qualitative research into women’s experiences has found that they may report trauma responses to births which are viewed by medical professionals as routine [10]; the subjective nature of the experience is the crucial factor [11]. Given that men are increasingly present throughout labour and birth [12] and that men’s post-traumatic responses influence those of their partners [13], understanding their experiences and responses is important.

Qualitative literature into men’s attendance at routine childbirth has identified that they experience uncertainty about their role in the labour setting, feelings of helplessness at the inability to support the partner in pain, but ultimately joy at the birth of a healthy child (e.g., [14, 15]); however, there is little research on the lived experience of men who have found attendance at childbirth traumatic. Nicholls and Ayers [16] studied the impact of PTSD on couples’ relationships, which included three men who had PTSD following childbirth. They found factors about the birth itself – such as that perceived lack of control over the events taking place - and a lack of care from staff contributed to the experience of trauma and that couples’ relationships were negatively affected by the impact of the birth events.

White [17] attempted to explore men’s experience of PTSD after childbirth through the narratives of fathers who had witnessed a traumatic birth, finding that men felt alienated through being “a spectator”, rather than having a role in the birth, and excluded by the actions of staff. Men reported feeling very distressed during the birth but tried to keep this hidden. The experience had an impact on the subsequent sexual relationship with the partner, which was described as “sexual scarring”. White did not measure or screen for post-traumatic stress symptoms. Furthermore, there was little information on the wider impact of the experience and how men tried to cope with the trauma of birth.

The limited literature in this area indicates that some men may experience symptoms of post-traumatic stress as a result of childbirth. There is very little research on the experiences of men who themselves found childbirth traumatic, regardless of whether they develop symptoms of post-traumatic stress. This study aimed to explore this phenomenon. The particular areas of investigation were: the factors that contributed to making the experience traumatic; the impact of the experience on the fathers in terms of their behaviour, relationships and how they tried to cope with the trauma; and what they believed could have supported them through the experience.

## Methods

### Setting and participants

The study took place in the UK. Participants were recruited via an advertisement displayed on the Birth Trauma Association website; in a newsletter of the Fatherhood Institute and on two internet forums: www.dadsnet.net and www.mumsnet.com.

### Inclusion and exclusion criteria

Inclusion criteria were that participants were: resident in the United Kingdom; aged 16 or older; had been present for the birth of the child; and described a trauma response to the birth of “fear, helplessness or horror” [18]. Exclusion criteria were: death of the partner or baby and infants who had spent more than seven days in neonatal intensive care following birth.

### Measures

The Impact of Event Scale (IES) [19] measures symptoms of traumatic stress. Possible scores range from zero to 45 with higher scores indicating greater severity of traumatic stress symptoms. The questionnaire assesses symptoms across two dimensions: intrusion and avoidance. The IES has been demonstrated to have good reliability (intrusion mean α = 0.86; avoidance mean α = 0.82) and validity [20].

### Ethics

Ethical approval for the research was granted by the University of Liverpool Research Ethics Committee.

### Procedure

Participants who responded to the study advertisement were sent a standard email response with the participant information sheet and a web link to access the online part of the study. Participants who accessed the website were presented with the participant information sheet for a second time before being asked to complete a consent form. They confirmed they met the inclusion criteria, including having experienced the birth as traumatic which was assessed with the question “At some point during the childbirth I experienced feelings of intense fear, helplessness or horror”. Participants who met the inclusion criteria and consented to take part then progressed to the IES [19]. This was included as a measure of current post traumatic stress symptoms and used to describe the sample. They were asked to complete this with regard to the experience of childbirth. Participants were then asked to provide a telephone contact number and preferred days and times for a telephone interview.

Interviews took place between January and May 2014. Participants were asked to confirm they had understood the participant information; reminded of their right to withdraw and asked to confirm that they wished to continue with the study. They were also given an opportunity to ask any questions. Interviews then proceeded following the semi-structured schedule and were audio recorded. The questions were:

- What was your experience of being at the birth of your child?
- What difficulties, if any, have you experienced following the birth?
- In what ways, if any, have these difficulties affected you and your partner?
- What things have you tried to help you manage how you’ve been feeling?
- How has your relationship with your partner been since the birth?
- How has your relationship with your child been since the birth?
- Is there anything that could have made your experience less difficult?

Two additional questions were added to the schedule after the first four interviews:

- What has it been like talking about your experiences?
- What motivated you to take part in the study?

### Analysis

Eight of the 11 interviews were transcribed by JE; the three remaining interviews by a professional transcriber. Interviews were analysed using Template Analysis, a form of thematic analysis, which can be adapted to suit the epistemological stand point of the researcher and incorporates elements of top-down and bottom-up analysis [21]. This method was selected over Interpretative Phenomenological Analysis (IPA) as it has a greater focus on between-case analysis and allows the researcher, through the use of a priori codes, to acknowledge specific areas of interest prior to beginning analysis.

Template analysis differs from other forms of thematic analysis in that it allows an outline template of key areas of interest, used to guide the analysis. The outline template is applied to the data and revised to incorporate new emergent themes, which are not already captured within the template. Subthemes are entirely populated by emergent data. Through this iterative process - which can involve the deletion or combination of information in the outline template - the final template, representing all the data, is developed.

Template analysis is concerned with the relationships between codes and themes, which is represented in the hierarchical structure. Using these higher-order themes as a guide, the interview transcripts were analysed for emerging sub-themes. Each interview transcript was read several times to become familiar with the data. Transcripts were then annotated with emerging codes which were added to the template.

**Fig. 1:**
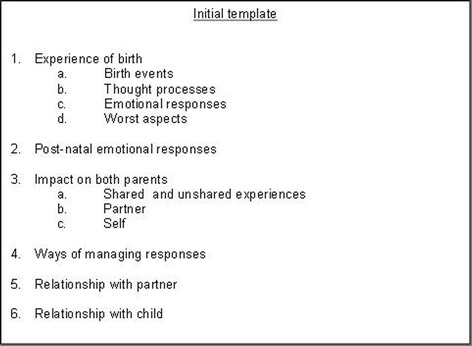
Initial template. Initial template based on interview schedule

An initial coding template following the structure of the interview guide was developed (Fig. 1). Transcripts were annotated with emerging codes which were added to the template. JE and PS read and coded the first three transcripts independently before comparing the analyses. Both researchers met again once all of the transcripts had been analysed to discuss the relationships between themes and the organisation of the final template. The template was revised throughout this process, with additional themes and subthemes inserted, deleted or collapsed under a new heading as the analysis progressed until the final template was developed.

## Results

**Fig. 2:**
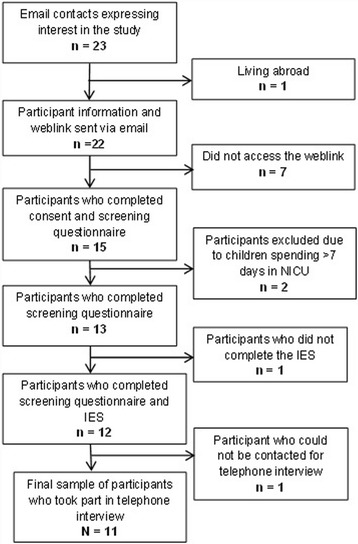
Flow of participants through the study. Response rates of participants throughout recruitment and interview process

Eleven men, aged between 27 and 45 years old *(M* = 36.36 years; *SD* = 4.63), participated in the study. Figure 2 illustrates the flow of participants. Interviews were between 28 and 87 min in length. All participants were first-time fathers at the time of the birth they had found traumatic; two men had gone on to have subsequent children. Seven were married, three were cohabiting and one man was engaged. All were employed. Two men had received previous treatment for depression, including medication and talking therapies. Both of these men had also accessed support from their GP following their childbirth experiences. Time since birth ranged from 2 months to 6 years.

**Fig. 3:**
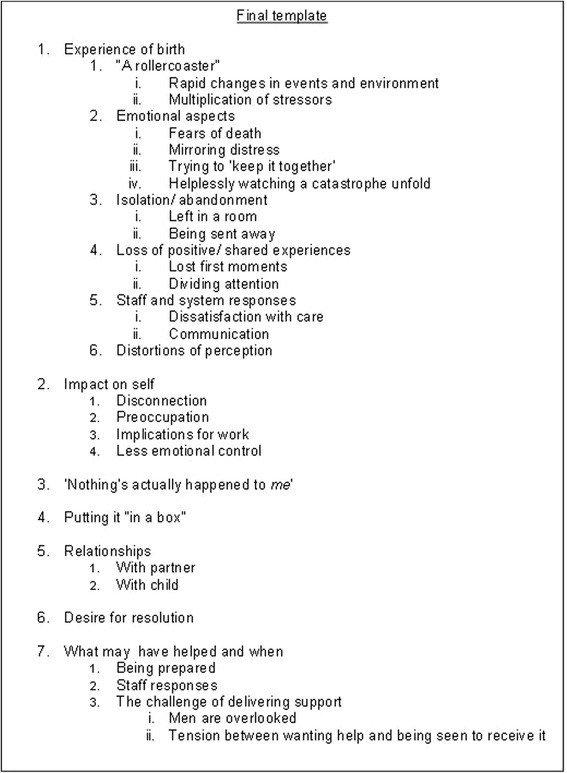
Final template

The final template is illustrated in Fig. 3. Seven higher-order themes were identified: experience of birth; impact on self; “Nothing’s actually happened to me”; putting it “in a box”; relationships; desire for resolution; and what might have helped and when. Four themes from the interview questions formed the outline template: experience of the birth; impact on the father; relationships; and what might have helped and when. The other three higher-order themes – nothing’s actually happened to me; putting it in a box; and desire for resolution - were entirely emergent. All of the subthemes were emergent. Care was taken to look for disconfirming evidence in the data and where this was found it is highlighted.

All participants described births in which complications had arisen (see Table 1). Table 2 illustrates participants’ responses on the IES. All but three respondents showed probable clinically significant symptoms on at least one dimension of childbirth related intrusions or avoidance [22].

**Table 1:** Birth details and IES score

Participant	Single/multiple birth	Mode of delivery	Pain relief	Complications	Total IES score
F1	Multiple	Emergency c- section	Spinal anaesthetic	Haemorrhage	44
F2	Single	Vaginal; forceps delivery	Gas & air; local anaesthetic	Episiotomy; anaesthetic administered incorrectly.	28
F3	Single	Emergency c-section	Epidural	Lost pessary; venflow administered incorrectly; ruptured retained placenta	35
F4	Single	Vaginal; forceps delivery	Gas & air; epidural	Haemorrhage	22
F5	Single	Emergency c-section	Epidural	Haemorrhage; hysterectomy	29
F6	Single	Emergency c-section	Epidural	Epidural not fully effective; lost swab	0
F7	Single	Ventouse	Gas & air	Ventouse; umbilical cord around baby’s neck	7
F8	Single	Emergency c-section	Anaesthetic	Premature	7
F9	Single	Vaginal delivery	Not known	Haemorrhage	27
F10	Single	Emergency c-section	Epidural	Abrupted placenta & internal bleeding	41
F11	Single	Emergency c-section	Pethidine	Haemorrhage	27

**Table 2:** Impact of Event Scale scores with subscales

Participant	Total IES score	Intrusion subscale	Avoidance subscale
F1	44	29	15
F2	28	6	22
F3	35	11	24
F4	22	8	14
F5	29	11	18
F6	0	0	0
F7	7	4	3
F8	7	1	6
F9	27	13	14
F10	41	15	26
F11	27	10	17

### Experience of the birth

The fathers’ narratives indicated that they found the experience of being present at birth one of constant shift and change, both in terms of situational aspects and emotional content. Their descriptions were frequently vivid and detailed. One father recalled how his wife’s blood “splashed up and hit the surgeon in the face”, likening the scene to an “abattoir” (F1). Another spoke of “the scream that came out of [his wife’s] throat” (F2); whilst another father recalled the contrast of holding his new born child in a room full of medical equipment with “blood dripping onto the floor” (F9).

#### “A rollercoaster”: rapid changes in events and environment, and multiplication of stressors

Most fathers referred to rapid shifting of events and emotion during the birth. These created an impression of the birth as “a rollercoaster” (F10), comprising two main aspects: the suddenness and speed of situational changes and accumulation of stressful events. Several fathers described thinking that the labour would go well and without difficulty; others had anticipated that the birth may be more complicated but felt unprepared for the scale of difficulty. Lack of preparedness for a difficult birth meant that the suddenness with which events changed was key:

“y’know, the first at least 24, maybe even 36 h or whatever, was absolutely straightforward and happy and relaxed and y’know we were all feeling very well prepared for what was coming. And then, and then that last hour, it just descended from being very straightforward to very traumatic very quickly. Erm, in a way that I couldn’t have envisaged even half an hour before” (F6).

The influx of additional medical staff with “more and more people turning up” (F3) to the labour room was recalled in many of the narratives as a sign that the situation had become urgent and often seemed overwhelming. The speed with which events took place contributed to a sense of disorientation and uncertainty:

“I just remember [the consultant’s] words……..’We’ve got to have it out now’ and it was all ‘Bang!’ and suddenly – I know it sounds daft but not being up close and personal with the NHS you’re limited kind of and I try and avoid those kind of soaps on TV - but it was like dramatic, everything happened. We suddenly then we were in a, in a, two minutes later we were in a delivery room and there’s a caesarean going on” (F10).

Several fathers described continual fluctuations of experience, from thinking that “we were over the worst of it” (F3) and “on the home strait” (F10), when the baby was born or the labour seemed to be under control, to then experiencing further stressors with concern about the health of either the baby or their partner. This short-lived relief and the cumulative effect of stressful events and shifting experiences was illustrated by one father:

And it’s like this pull, push, pull, push, pull, push, in, out. Over, back of what’s happening; what’s not happening; what could happen; what might; what you thought would happen; what’s not (F1).

This rollercoaster effect was a thread running throughout many of the narratives creating a level of uncertainty, anxiety and helplessness with which the fathers found it difficult to cope.

All the kind of stuff that went on before that and all the stuff that went on after that might not have been that significant in themselves but when it’s all put in the mix together it just multiplies up (F3).

Not all of the stressful events seemed traumatic in themselves but the multiplication of stressors led to distress for the men. Both situational and emotional aspects contributed to this. The partners of four men experienced medical mistakes such as pain relief being administered incorrectly, which added to the pain experienced and increased both the mothers’ and the fathers’ stress. Others recalled instances of lack of care from staff, or the experience of waiting for news and information.

#### Emotional aspects: fears of death, mirroring distress, trying ‘to keep it together, and helplessly watching a catastrophe unfold

Men described strong negative emotions during the birth which they tried to contain in an attempt to protect their partners from further distress. Ten of the 11 men described fears that their partner or baby would die:

“You know, at one stage I was thinking, ‘sh**, I’m going to bring up a child on my own’” (F11).

“I remember just sort of having a sense of my life, my whole life, sort of in some kind of pivot, I suppose. And just thinking, d’y’know, ‘Whatever happens here’ y’know, ‘is this the moment where, y’know, my, my partner dies and I, I have that and I have a son and I’m going to be a dad that brings this boy up by myself?’ (F9).

The strength of feeling was illustrated by the powerful language fathers used, referring to themselves as “terrified” (F9 and F10). These fears often arose from the men’s perception of what was happening:

“from what was going on, y’know, the amount of people in the room and what she was looking like and what they were doing and all that kind of thing, I was thinking “oh god, this is 50/50” (F4).

The pain of the woman and her suffering had a direct effect on the man as his distress mirrored hers:

“the more that she was obviously in distress the more I became distressed on her behalf” (F6);

“And the really traumatic part was just, the sort of, the scream that came out of her throat was absolutely horrible to…it just really hurt to see her in so much pain.” (F2).

The experience for one father of hearing “for an hour my wife screaming in the room next to me and I wasn’t able to do anything about it” (F3) was the most traumatic aspect of the birth.

Seven fathers referred to “trying to keep it together and be strong for [partner]” (F8), although this belied the emotion they were really feeling: “I wasn’t calm in my head but outwardly I was” (F7). This seemed to be motivated by the belief that, for the woman, seeing her partner upset was “not going to do her any good at all” (F9). Most of the men tried to hide their feelings but some were so overwhelmed by the experience that they could not maintain this façade and “broke down” (F11). Others were able to contain their emotion during the birth but became extremely distressed when on their own and “just broke down in tears” (F5).

A “feeling of utter helplessness” (F7) was a key theme, with one father likening the experience to “watching a, yeah, a car go off a cliff and, you know, you’re just slightly too far away to actually do anything about it and you just sort of stand there and watch it all happen” (F4).

Perceiving themselves unable to do anything practical, alongside uncertainty from a lack of information and understanding of what was happening, also left the men feeling helpless:

“I felt helpless and I thought ‘I don’t know what’s going on’” (F10).

“at that point I didn’t know what was going on. I didn’t know if she was in, y’know, was in physical danger, if my baby was, erm what was actually happening. I didn’t have a clue.” (F3)

This was in contrast to their usual experiences of feeling in control: “I spend a lot of my life being in control of stuff and looking after stuff and managing stuff……I was in a situation where I was, er, I felt like I was…totally out of control” (F8).

Consequently, the men had to put their trust in the health professionals, which gave them a sense of unease:

“There’s just nothing you can, you can do to sort of change what’s gonna happen and sort of thing. It, it’s up to somebody else to sort of sort out, or not as the case may be” (F4).

Linked to the feeling of helplessness was the fact that the men were very conscious of the medical activity in front of them, the actions of staff and some of the visual images they were confronted with; in contrast sometimes to their partners:

“but at the same time I’m sitting there and *I’m sitting there*. There’s no drugs in me, I’m not anaesthetised in anyway” (F1).

#### Isolation and abandonment: left in a room and being sent away

For several fathers, the experience of being present at the birth involved a period during which they felt abandoned or isolated. Men often found themselves alone either while their partner was being prepared for a surgical birth or when she was being attended to by medical staff after the birth. The men gave stark examples of how isolated they were at times:

Suddenly, erm, I’m just handed the baby and, er, and [partner] and these ten or twelve people shoot out of the room……literally everybody left (F9);

So I was just then left in that room on my own and I was in there for, I was in there for over an hour (F3);

This isolation increased anxiety and uncertainty about what was happening, intensifying the men’s fears that something was wrong:

They took me and, er the babies back to a room whilst they said they were going to clean [wife] up a bit and what have you…It took about..it took her about half an hour then to reappear so …I thought “what the hell is going on here?” (F1).

“So I was a little bit lost in, well, just this big empty room at the end of the day and I was just like ‘oh my god, what’s going on? What’s going on with [son]?’” (F5)

For some, a sense of abandonment was felt keenly when they were told to leave the hospital after visiting hours and were left alone but very upset:

“When they sent me home at two in the morning and I remember just sitting in the kitchen for hours in bits” (F9).

“’There you are. Go. And don’t come back in the morning til ten o’clock.’, I found that so difficult” (F7).

This was sometimes compounded by the emphasis from staff that facilities were for the mother and child only, not for men. A notable exception was a man whose wife was very ill after childbirth and who was allowed to stay with her in hospital; being treated almost as another patient.

#### Loss of positive, shared experiences: lost first moments and dividing attention

A consequence of the nature of many of the births was that some of the first moments with their child, which the fathers had envisaged being shared, joyful events, were lost. They also found themselves in the position of having to negotiate dividing their time between their partner and baby.

Five men made specific reference to the loss of anticipated positive experiences as a result of what happened during the birth. For some, the situational aspects and stress of what was happening left them with a sense of regret that they had missed out on the anticipated “magic moment” (F3) of birth. For others, the medical attention needed by their partner meant that some of the things that they had expected to do with them were done alone; “I just anticipated that we’d do it together” (F9). This was made more difficult when the men were aware that their partner would have wanted to do things such as change the first nappy and give the baby the first bottle. For some men their partner’s physical condition after the birth meant that they had to continue as the primary carer for the baby for some time after birth, which was a positive experience but one “tinged with guilt” (F10) that their partner would have wanted to do it.

Fathers spoke of “flitting between” their partner and child (F10) and feeling that they were “torn between two posts” (F3). One father reported that he “couldn’t really comfort [partner] because I was holding the baby” (F5), whilst another did not want to leave his wife in theatre to go with his new-born daughter (F11). Although most of the fathers felt that the loss of shared experiences was a negative consequence of the birth, one man stated that although “there was no enjoyment of the moment” he did not think that this was a problem because “you can’t miss what you’ve never had” (F10). In addition, one man acknowledged that it was a “special thing…to be there for [baby]” (F9).

#### Staff responses: dissatisfaction with care, communication

For some men, interactions with health professionals contributed to their distress during the birth. Five men described difficulties with staff due to feeling “dismissed” or through perceived lack of care: “I didn’t really feel like we were getting much help” and the midwife was “horrible” (F3), causing this to be central to finding the experience traumatic. One father expressed anger towards staff at their perceived negligence:

“that became very mixed up with emotions of sort of anger, like ‘what on earth d’you think you’re doing?’ y’know? ‘Why…how could you possibly have been so negligent to do that?’” (F6).

Lack of sensitivity in the way staff spoke to either of the parents also caused distress for the men. One father described himself as “destroyed” (F7) by a comment from the midwife and another felt his partner’s difficulties were not acknowledged by staff, telling her “we have women who’ve had C-sections in here and they’re up and about” (F9).

Some men were very angry about how they had been treated; others recognised the difficulty for staff of attending to them and giving information whilst they had a job to do in looking after the woman and child. However, they would have liked more communication. One father recalled vividly the sight of his daughter being “smuggled away” (F1) by staff as they tried to revive her, without acknowledging to him that she had been born:

Nobody said anything. You know, that’s…even if the child was born and was dead I would’ve expected them to say something. Not just disappear around the corner. And, and because they didn’t, you kind of have to…where I was at the time I think “There must be a reason they’re not saying something. There must be a reason”.

Other men expressed the difficulty of “waiting and waiting and waiting for something, for some information” (F8) and having “no explanation of anything” (F3) during the birth. Participant eight understood this as resulting from the fact that “this is very mundane for [staff] and they understand what’s happening and you don’t”.

In the absence of communication from staff, some men tried to infer from body language or their surroundings what was going on; including “scanning for clues” (F8) in the operating theatre or trying to read the body language of the medical staff. Some gave positive descriptions of how staff had communicated with them through the birth, which gave them reassurance, but also reflected that there were times when they would have liked to have known more about what was happening.

#### Distortions of perception

Several men reported changes in perception and a “heightened awareness” (F1) as the events of the birth took place. Most common was a sense of everything taking a long time; it “felt like forever” (F5); “it felt like about 3 days” (F3). This was usually in the periods when fathers were waiting for information and separated from their partners. Others reported the experience as “surreal” (F10) and that everything happened in “a blur” (F11) and “didn’t really register properly” (F4). Another described “the feeling of being removed slightly from the reality but still…you’re there; it’s very real. It’s so real that it’s unreal” (F1). Some of these effects continued to impact upon the men after the birth.

### Impact on the father

Most fathers indicated that the birth experience had had some impact on them, to greater or lesser degrees. For some their distress continued to be acute, whilst for others the impact had lessened over time.

#### Disconnection

For some of the fathers, the feeling of disconnection which had begun with the distortions of perception during the birth continued afterwards. The degree of disconnectedness ranged from being “completely shocked” (F7) and “stunned” (F1), to going onto “autopilot” (F10), to the man who “went off into some weird twilight zone kind of thing, I think…I sort of, not zoned out for 6 months but sort of…I had an odd reaction” (F4). This was in part related to how the men tried to cope with the enormity of the experience of birth.

#### Preoccupation

Preoccupation with the events of the birth continued in the weeks, months and even years afterwards; being “still fresh in my mind” (F11). Rumination was a feature for some men who found themselves “going over and over” (F3) what had occurred, with one father even trying to imagine what pain his partner must have been in. External stimuli, such as television programmes and stories of babies being born, triggered their memories of the birth and as a consequence they tried to avoid them. Some had flashbacks to the birth or relived it in their dreams. At the most severe end of this continuum, was a father who described such intrusive thoughts of the birth that, a year on, he never went to bed, instead watching television on the couch until he slept, “because even if I go to bed, even still the events of [the birth] go round and round and round in my head” (F1), preventing him from sleeping. Two fathers identified that the birth had had a delayed impact on them. They were both men with a previous history of depression, which after a period of around 12 months of coping, they felt was returning.

#### Implications for Work

For some men, the birth experience and its personal consequences adversely impacted up on their work. One father who described himself as previously very dedicated to work explained that he had had his “least productive twelve months” (F9) since first being employed; another had reduced his workload due to difficulties concentrating, and one man had taken a sabbatical from work “I kind of got to the point that I said, look I need to stop work for a bit and…take some time out” (F8). In contrast, participant four took refuge in his work; attempting to retain some normality through the belief that “I’ll just go back to [work] and everything will be fine”; another found work a helpful distraction.

Some men commented on their reluctance to discuss their experiences, particularly with their colleagues: “We’re like a lot of males, male-orientated and, sort of, like you don’t want to show your emotions and like it’s all a bit of a laugh. You make a joke of things all the time” (F5). The expectation that others may not understand or would dismiss their distress also affected how fathers shared their experiences of the birth.

There’s no point going to work and saying “My head is all over the shop because of what happened back here” and, y’know, and it’s like you can’t say that to somebody who’s looking at the photo of your two beautiful new-borns. And they’re looking at you and “you should be pulling yourself together, forget about that now” (F1).

#### Less emotional control

Men described feeling “much more emotional” (F5) and that this was unpredictable. Often they were unsure whether this was an effect of being at the birth or a normal response to becoming a parent but it was usually felt to be inappropriate. Participant ten recalled becoming emotional over a story he heard on the radio, explaining “that made me kind of go into tears. I thought, that’s not, that’s not right, I mean it’s a lovely story but…”.

### “Nothing’s actually happened to *me*.”

Although for many of the men the experience of birth had a very significant impact on them, fathers indicated that they did not feel that they had a right to be affected because “nothing’s actually happened to *me*” (F5); dismissing the experience of witnessing and being part of the birth because it was emotional, rather than physical:

“I felt guilt…that I was feeling traumatised when, you know, obviously I hadn’t really gone through anything” (F11).

Intertwined with this idea was the concept of masculinity and what the fathers expected of themselves. As one man explained, “someone had to be the strong one” (F8) and

“there’s no room, if you like, for me feeling sorry for myself, or, or having time to be a patient” (F1).

Fathers felt that as they had not physically given birth it was their responsibility to “get on with it” (F1). These beliefs dictated the coping strategies that the men employed to deal with the effects of the birth.

### Putting it “in a box”

The men’s coping strategies seemed to be dictated by their beliefs about acceptable masculine roles such as stoicism, keeping emotions to themselves and being strong for others. Avoidance was the dominant theme and was evident at some level even for the men who spoke of their difficulties as resolved. Fathers referred to having “bottled it up” (F8) or “putting it in a box” (F1) and not “wanting to get into it too much” (F10). This occurred along a continuum from avoiding thinking or talking about the birth to the father mentioned previously who described himself as in a “twilight zone” (F4) for several months after the birth.

This also related to the idea of ‘getting on with it’ which included elements of avoidance such as hiding feelings from their partners because “I’m a big lad, I can look after myself” (F1) and not wanting their partner to “know how scared I was that night” (F5). Fathers tended to focus on the present and getting through each day, at the cost of addressing their emotions about the birth. However, most did acknowledge that, as a coping strategy, this had potential negative consequences. One father who described himself as “falling to pieces” as a delayed impact of the birth explained

“I kind of wonder if that’s because it had just been bottled up and held and like ‘come on, get on, get on, get on, get on, deal with it’” (F8).

There were some exceptions to avoidance. One man had forced himself to think about the experiences as a way of dealing with them and felt that they were now resolved. Another tried to watch programmes about birth to help him deal with his feelings. Another described an almost desperate search for some context in which to understand his experience, “anything that could help me process what, you know, that, that day” (F11).

Two fathers had sought help for their distress. Interestingly, these were both men who had a previous history of depression. Both described being aware of the signs that they were having difficulties with their mental health and both had also had some form of counselling or talking therapy on previous occasions. However, both had reached a significant level of distress before seeking help.

### Relationships

The experience of the birth had various consequences, both positive and negative, on the men’s relationship with their partners and children.

#### With partner

For some men the experience of the birth had a positive impact, including a deepening of feeling for their partner and the relationship taking on a new dimension:

“I think her, her having such a kind of intense experience, it also brought home to me the fact that she was not just my wife any more, she was also the mother of my child” (F6).

For others, the impact of the birth and how the men tried to cope with it acted as a barrier in the couple’s relationship. One man’s attempts to cope with his distress by withdrawing and hiding his emotions resulted in his partner perceiving that he was “blasé……that I didn’t care too much about what had happened” (F5). Other partners felt unsupported and, in some cases, the level of disconnection was such that the man felt there was little relationship left with his partner.

The partner’s response to the birth also affected how the man felt. One explained that “it helped me to see her dealing with it” (F2), whilst for others the on-going emotional or physical effects of the birth on their partner prolonged their distress and it was “difficult to deal with her dealing with it” (F3). In some couples, the men and their partners had very different mechanisms for coping with the experience and some of the men commented on the very different perspectives that they had each had of the birth as “she didn’t see it from this angle” (F10).

For three men the experience had affected their feelings about future pregnancies. For one, “talk of another child means it’s all coming back” (F11); other fathers doubted their ability to cope:

“It made me wary of the process of the delivery and where it might put me. And if I was to have that experience again, would it be worse? Could I cope?” (F7).

This had impacted on some of the relationships in that their partners had wanted to have more children sooner.

#### With child

All bar one of the men described that, despite the traumatic experience of the birth, their relationship with their child was, for the most part, positive. Some believed that the difficult experience of the birth and the effects on their partners had a positive consequence on increasing their bond with the baby, particularly if they had had to take on the role of primary carer for a period after birth; “I got very good at being hands on and doing it all right from the start” (F10).

One exception was father 4, who felt that his relationship with his child was “uneasy”, which he attributed to the impact of her birth on him. He described himself as “zoned out” through the early months of her life and thought this was the cause of their lack of close relationship. The impact for him was long-lasting as his child was almost seven years old.

### Desire for resolution

A minority of the men felt that their difficulties with the birth were resolved; however for most there was a sense that something endured which was yet to be addressed. For some, the fact that things had still not “got back to normal” (F1) and that the effects of the birth were “still front and centre in our life” (F3) compounded their difficulties. Others felt that, although the impact on them had reduced, there was a lingering effect, “a cloud” (F10), and that the experiences still needed “straightening out in my head” (F9).

### What might have helped and when

Two main aspects could have reduced men’s distress: being prepared and the responses of staff and the health system. A third theme arose from the men’s comments: the need for support yet the difficulty of providing it to men, who are loath to be seen seeking help.

#### Being prepared

Three men suggested that being aware that there could be difficulties would have reduced their distress during the events of the birth. Most had not anticipated anything other than a straightforward birth, suggesting that the knowledge that labour and birth is “not risk free” (F5) could have helped. An antenatal class demonstration of a surgical birth had helped one father feel reassured when he came to experience one. However, one man thought that “nothing can prepare you for it” (F8).

#### Staff and system responses

Six men indicated that there were things that staff could do to help them through the process. They generally thought that better communication would have been helpful although they acknowledged the difficulty of doing this during the labour. However; most felt that after the birth “just having someone to talk to” (F7) and “anything that could maybe help me process what, you know, that, that day” (F11) would have been helpful. Following a difficult birth, fathers also desired acknowledgement from staff that it “wasn’t normal” (F1) and could be difficult for the man. Two fathers had had a follow-up meeting with members of the medical teams involved in the birth; another had decided with his partner to ask to view the medical notes in an attempt to help him process what had taken place during the birth. It seemed important to the men to be able to gain some context for their experience in order to be able to deal with it.

#### The challenge of delivering support: men are overlooked; tension between wanting help and being seen to receive it

There was a tension and contradiction in the men’s responses to the question of what would have helped them, which was that they expressed that there should be more support for fathers but also that men would be unlikely to access help.

Most men seemed prepared for the attention during birth and immediately afterwards to be “all rightly on the mother” (F11); however, they also thought that men were “tertiary” (F10), often “overlooked” (F8) and “an afterthought” (F5) and that after the birth there was “nothing for the chap. Erm, and I felt I needed it far more than [wife] did” (F7).

Despite believing that men should be supported, the fathers conveyed that men “don’t like to show weakness” (F11) or be seen to access help. One father explained that he would not have taken up any support offered at the time because “I’m a man” (F4), whilst another maintained that even when he was asked how he was feeling “you say you’re fine, because there are two people who are far more important” (F10). Some men commented that taking part in the study was useful because “talking about it helps” (F11), particularly “in a completely anonymous way” (F10). An opportunity to talk about their experience was often part of the motivation to participate. One father suggested that taking part had validated that his experience was difficult and upsetting as, “you’re allowed to be emotional about it because someone understands that it does affect the partner as well” (F5).

## Discussion

This study provides new information concerning the nature of men’s experiences when they have found being present a childbirth traumatic; what factors contribute to the experience of childbirth as traumatic; and the consequences for them and their families. In summary, men who experienced childbirth as traumatic described this as a rollercoaster of emotions with events building on each other. They were fearful of the death of their partner and or baby, mirrored their partner’s distress but tried to present a controlled exterior whilst feeling they were helplessly watching a catastrophe unfold. They felt abandoned and isolated - often being excluded - and that staff did not inform them or treat them with sensitivity.

They felt they lost the anticipated positive shared experiences with a newborn. In terms of their relationships with their partners, through respect for what she had endured, on occasions there were positive impacts and the relationship with the child was not generally adversely affected. However, postnatally the experience of birth continued to resonate, leading to disconnection, rumination and continued distress. Men tried to block and avoid these responses feeling that they were unjustified as ‘nothing had happened to them’. They recognised the need for help but were reluctant to seek this. All of the men met criterion A for PTSD [23]. The IES [19] is not diagnostic, therefore it is not possible to state whether any of the participants would meet the criteria for PTSD; however; most participants reported probable clinically significant symptoms and, regardless of symptomatology, all men reported distress that had subsequent impact on their lives.

The men’s experience of the birth as “a rollercoaster” resonates with other findings on fathers’ experiences of non-traumatic childbirth [e.g., 14, 15]. However, the fathers in the current study did not experience euphoria at the end of the process; for them, layers of stress accumulated throughout labour and birth and, for some, after birth. This is an important distinction, particularly when considered within a stress and coping framework (e.g., [24]). According to Lazarus [25], stress occurs when “there are demands on a person which tax or exceed his adjustive resources” and is mediated by appraisals of threat. The accumulation of environmental stressors during the birth and the frequent shifting of events and emotional experiences contributed to fathers’ threat appraisals, such as fear of death. At the same time, the nature of the birth situation meant that the men were unable to use typical coping resources, such as taking control and problem-solving. This combination of factors contributed to the experience of extreme stress which, for some of the fathers, continued for a long period of time.

Men have been found to favour problem-focused coping strategies for managing stress, which usually rely on taking control and action [26]. Their frustration with lack of communication from staff and the search for information about what was happening reflects this. However, in most cases, their attempts to use these strategies seem to have been thwarted, leaving the fathers feeling out of control during the birth; something which Nicholls and Ayers [16] found to be associated with PTSD after childbirth.

The distortions of perception experienced by some fathers during the birth may indicate peritraumatic dissociation which, in women, has been shown to be an indicator of subsequent postnatal PTSD [27]. It is not possible to infer from one study of this size but the experiences the men described indicate that further investigation of these phenomena in fathers is warranted.

The isolation and abandonment described by the men is also of interest. Men can often feel excluded to some degree during childbirth [28–30] but the present study suggests that this may be far more important than has perhaps been recognised as it intensified the fathers’ distress and anxiety. This is a relatively unique finding in the literature with one other brief report on fathers of infants in neonatal intensive care mentioning this profound sense of abandonment [31]. The finding suggests a role for maternity services in providing additional support for fathers at these times.

The birth experiences described had a significant impact on the fathers’ lives and functioning. Experiences of disconnection and preoccupation with the events are consistent with post-traumatic stress, as is avoidance, which was the primary coping strategy adopted by most of the men. Ideas about masculinity seemed to influence the men’s strategies for managing their distress. Pleck’s [32] gender-role strain paradigm states that in situations of conflict between expected masculine roles and the demands of other roles dysfunction strain may occur. Conflict between the internal need to remain ‘manly’ and stoic in the face of the accumulation of stressors may have led to the men coping through avoidance; but this had significant consequences for some of them. Ehlers and Clark [33] suggest that avoidance of stimuli associated with trauma reinforces a person’s negative thoughts and beliefs about their ability to cope, or the consequences of the trauma, such as their fears of continuing threat. Avoidance prevents the individual from incorporating the trauma memory into its context and reducing the appraisals of threat.

The ideology of masculinity is particularly pertinent to the men’s perception that “nothing’s actually happened to *me*” and the view that being affected by the emotional aspects of the experience was in some way unjustified. This seems to have established how they would cope with the experience afterwards, leading to the avoidance strategies adopted. White’s study [17] demonstrated that men tried to avoid showing their distress after the birth; what this study adds is that some men do not view what they have been through as something that should cause distress. They seem to invalidate their own experiences, which subsequently impacts on whether or not they access support. Despite this, most of the men acknowledged that they were distressed and desired a resolution to the experience.

Seeking support for problems that are perceived to be unusual, or non-normative, poses a greater risk to self-esteem than problems that are viewed as normative [34], which could explain why some of the men, though clearly distressed, were reluctant to acknowledge their difficulties to others or seek help. What seems poignant is that this dismissal of their experience and the coping strategies that the men employed are likely to extend, rather than resolve, their difficulties with the birth experience. Ehlers and Clark’s [33] cognitive model of PTSD posits the importance of integration of trauma memories, which strategies such as thought suppression and avoidance, as used by these fathers, prevents. Furthermore, the tendency for men to avoid seeking help for either physical or mental health problems [34] is likely to prolong their distress. It may also increase the distress for partners, as several men acknowledged the negative impact of their feelings about the birth on their partners, which is consistent with the findings of Nicholls and Ayers’ [16] study with couples. It is, however, encouraging to note that almost all of the fathers felt that their relationships with their children were positive and had not been adversely affected by their experience of the birth.

### Clinical implications

The findings have implications for maternity services and how they meet the needs both of women and men within pregnancy, labour and childbirth. Within fifty years men have moved from being total outsiders to the birth experience to a position of being almost mandated to be present. This has been a seismic shift, yet the culture of maternity care has not adapted to this change. The view expressed by men that “nothing’s actually happened to *me*” is perhaps mirrored by the way maternity services treat them; paying relatively little attention to fathers, other than as a supporter for the mother. Men are also becoming parents and are exposed to everything that occurs during labour and birth therefore it seems reasonable to suggest that they should be treated as recipients of care in their own right.

Within the broader context, continuity of care has been shown to be an important factor in determining women’s experiences of childbirth (e.g., [35, 36]). Having both ante and perinatal support from the same midwife has been shown to promote a trusting relationship between the mother and midwife, which contributes to more confidence and less fear before and during the birth. Aune et al. found that fathers, rather than mothers, valued more highly the presence of a student midwife throughout the childbirth itself [35]. This practice of continued and consistent care may help to address some of the difficulties highlighted by men in the present study.

Men’s desire for preparation suggests that services need to acknowledge the different needs of fathers and mothers in pregnancy and birth. Men desired communication and information to help them contextualise their experiences. Antenatal care should include some information about what will happen during different types of births, including emergencies, in order to help provide this context. The study also highlights the importance of care for both parents, particularly during complicated births. Primarily, good care of the mother throughout the birthing process is likely to reduce distress for both parents; however, men and women have different experiences and perspectives through childbirth and there may be individual needs that should also be attended to, such as how fathers are supported during times that they cannot be present in surgical or emergency births. Fathers want to be near their families after distressing births [37] and report more positive experiences when they are able to remain in hospital [38]. This is not common practice in the UK but is something which should be considered in terms of supporting both mothers and fathers following childbirth.

The effect of cumulative stressors on the men’s experience suggest that it may be difficult for health professionals to pick up on fathers’ distress, as what staff might view as minor incidents could be having a far greater impact on the men within the wider context of the birth experience. In addition, men’s reluctance to show what they perceive as weakness in the situation means that it is unlikely they will ask for support. Discussions within antenatal classes about possible responses to the birth experience could help to normalise these for men and thereby increase the likelihood of accepting support if it is needed. Acknowledgement of the needs of fathers during postnatal Health Visitor checks may also legitimise for men that their coping following childbirth, as well as the mother’s, is important and can be discussed. This may also be important in assessing post-natal support for the mother, given that traumatic birth experiences can impact negatively on the couple’s relationship.

As mentioned, a father’s presence is now generally expected in modern western cultures; however, there is some emerging evidence to suggest that it can have a negative impact on the woman’s birth experience and some clinicians have argued against it as a practice (e.g., [39]). Given the potential negative impact of the childbirth experience on the father, a wider clinical issue is to consider whether men should be present at childbirth at all and whether the benefits outweigh the costs and for whom..Maternity services could address this through providing opportunities antenatally to discuss with families the pros and cons of fathers’ presence at the birth, thereby legitimising the option of not being present.

### Limitations

It was recognised prior to the research that telephone interviews may have limitations in terms of engaging participants and building rapport; however, several participants reported that the anonymity provided by a telephone conversation was preferable.

Most fathers described some form of obstetric complication during the birth. This likely reflects the recruitment strategy of accessing men via trauma-related and fathers’ websites. It may mean that fathers who have found birth traumatic for reasons other than obstetric complications may have been less likely to find and take part in the study. Furthermore, the clear message that men are very reluctant to either acknowledge their difficulties or access support, and the dominant coping strategy of avoidance, indicate that there are likely to be men whose experiences would be important to the study but who would not come forward to participate.

### Future research

Longitudinal studies with fathers to investigate the risk factors for developing mental health difficulties such as PTSD following child birth, the prevalence and course of the difficulties are required. Almost all of the men in this study experienced objectively complicated births suggesting that further research into men’s experiences of non-routine childbirth would be useful and may help to clarify the factors that contribute to subjective distress. Future research could include intervention studies into how information is provided to fathers and how they are prepared for birth and supported afterwards. Additional research into the nature of couple’s experiences of a traumatic birth may also help to elucidate interactions between how the impact of a traumatic birth on each parent may affect how the other copes with their birth experiences.

## Conclusions

This study adds to current literature that suggests being present at childbirth can be an extremely distressing experience for men and one which may induce symptoms of post-traumatic stress, which can be severe and enduring. It suggests that it is essential for maternity services to routinely attend to the needs of fathers in their own right before, during and after childbirth. This may include: father-focused information about the birth process and their role; provision of continuous support for both parents through the birth process; and, following birth, the opportunity for families to remain together in hospital. Masculinity ideology may act as a barrier to men accessing help; therefore support structures, such as post-natal reviews or health visitor checks on the father’s coping, should be in place as a matter of course for all fathers.

## Abbreviations

IES: Impact of event scale
PTSD: Post-traumatic stress disorder
